# Implementation of tumor-free and total Müllerian compartment resection techniques in robot-assisted radical hysterectomy: protocol for standardizing surgical procedures

**DOI:** 10.3389/fonc.2025.1598519

**Published:** 2025-10-06

**Authors:** Xinyou Wang, Jinming Zhu, Siman Li, Jing Na, Jun Wang, Shichao Han, Ya Li

**Affiliations:** ^1^ Department of Gynecology and Obstetrics, The Second Affiliated Hospital of Dalian Medical University, Dalian, China; ^2^ Dalian Medical University, Dalian, China; ^3^ Oncology Department, Affiliated Zhongshan Hospital of Dalian University, Dalian, China

**Keywords:** cervical cancer, radical hysterectomy, membrane anatomy, robot, membrane bridge

## Abstract

**Objective:**

The main objective of this study is to apply the tumor-free technique in robot-assisted radical hysterectomy to effectively prevent tumor exposure and dissemination during the operation. Meanwhile, this study aims to standardize and optimize this technique, thereby promoting its wide application in clinical practice and ensuring the stability and reproducibility of surgical outcomes.

**Method:**

The surgical indications for this study were as follows: patients with stage IA2, IB1, IB2, IIA1, and certain specific pathological types of IB3 and IIA2 cervical cancer(FIGO 2018). During the operation, suture suspension of the uterus was used instead of a uterine manipulator. Before incising the vagina, the vaginal orifice was closed. After completing the vaginal closure, the vaginal wall was rinsed with 42°C sterile distilled water. All surgical procedures followed the concept of embryonic compartment-based hysterectomy according to membrane anatomy, ensuring the integrity of the Müllerian embryonic compartment’s membrane structure.

**Results:**

Guided by the concept of membrane anatomy, robot-assisted radical hysterectomy facilitates bloodless surgery while improving surgical efficiency and precision through the simplification and optimization of techniques. Moreover, this approach maintains the integrity of the Müllerian duct embryonic compartment, thus preventing tumor spillage. When integrated with tumor-free exposure techniques, it offers cervical cancer patients the advantages of minimally invasive surgery, including faster recovery, reduced surgical trauma, and a lower risk of iatrogenic tumor dissemination.

**Conclusion:**

Employing robotic technology in conjunction with the concept of membrane anatomy during radical hysterectomy can lead to a more meticulous and precise surgical outcome. The application of precise surgical techniques not only facilitates the standardization and optimization of procedures but also minimizes patient trauma and accelerates recovery.

## 1 Introduction

The introduction of total mesorectal excision (TME), which involves high-resolution sharp dissection of the rectum and its associated mesentery guided by developmental anatomy, has substantially improved postoperative outcomes, reduced locoregional recurrences, and enhanced survival in rectal cancer surgery (1, 2).

Research by Höckel and Fritsch into the embryonic development of the female reproductive tract, particularly across distinct embryonic compartments, has revealed important implications for managing cervical cancer (3–5). Their work underscores the effectiveness of total mesometrial resection (TMMR) in achieving local tumor control without the need for adjuvant radiotherapy (6, 7), and has elucidated patterns of local spread in advanced and recurrent disease (8). These insights challenge current classifications of radical hysterectomy and criteria for adjuvant radiotherapy (9).

Several other groups have also reported on the feasibility and safety of this technique (10–13), though long-term data on morbidity and survival after laparoscopic radical hysterectomy remain limited.

Radical hysterectomy demands a high degree of surgical proficiency. Robot-assisted radical hysterectomy enhances operative efficiency and precision through stable three-dimensional visualization, articulating instruments, and tremor reduction. Under the guidance of embryonic compartment-based resection within membrane anatomy, the procedure involves separating and completely removing the Müllerian compartment from the hindgut-derived rectum, the ureteric compartment’s ureter, and the urogenital compartment’s bladder. Consequently, robot-assisted radical hysterectomy presents considerable technical challenges.

## 2 Indications

Patients with stage IIA2, IB1, IB2, IIA1 cervical cancer (staging is based on the FIGO 2018 classification system), as well as selected cases of IB3 and IIA2 cervical cancer with specific pathological features, were included. There was no radiological evidence of lymph node metastasis, as assessed by magnetic resonance imaging (MRI) or computed tomography (CT).

## 3 Perioperative considerations

Preoperatively, patients with cervical cancer often present with prolonged vaginal bleeding, discharge, and tumor ulceration, creating an environment conducive to inflammation and infection. To mitigate these risks, preoperative vaginal flushing with povidone-iodine solution for at least 3 days is recommended. This practice helps maintain vaginal cleanliness, reduces congestion in the parametrium and paracolpium, and ultimately facilitates surgical procedures while lowering the likelihood of postoperative infections.

During surgery, in cases of obesity or significant ureteral exposure, the consideration of D-J tube placement can help reduce the risk of postoperative ureteral fistula complications.

## 4 Method

### 4.1 Docking

**Figure 1:**
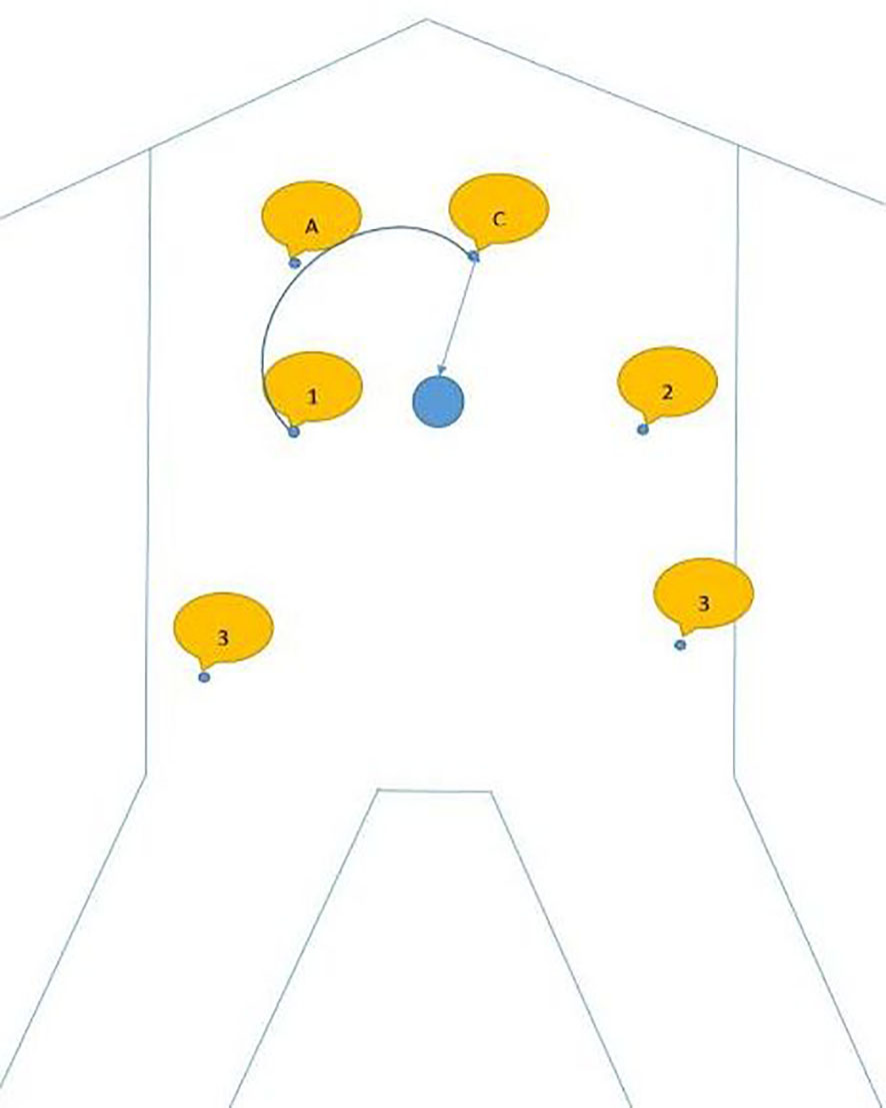
A shows assistive hole; C shows camera arm puncture hole; 1 shows puncture point for arm 1; 2 shows the puncture point for arm 2; 3 shows the puncture point for arm 3.

The specific docking method is shown in **Figure 1**, also can be referenced from the author’s previously published article on radical trachelectomy (14) (Tumai, **Figure 1**).

### 4.2 Uterine suspension

**Figure 2:**
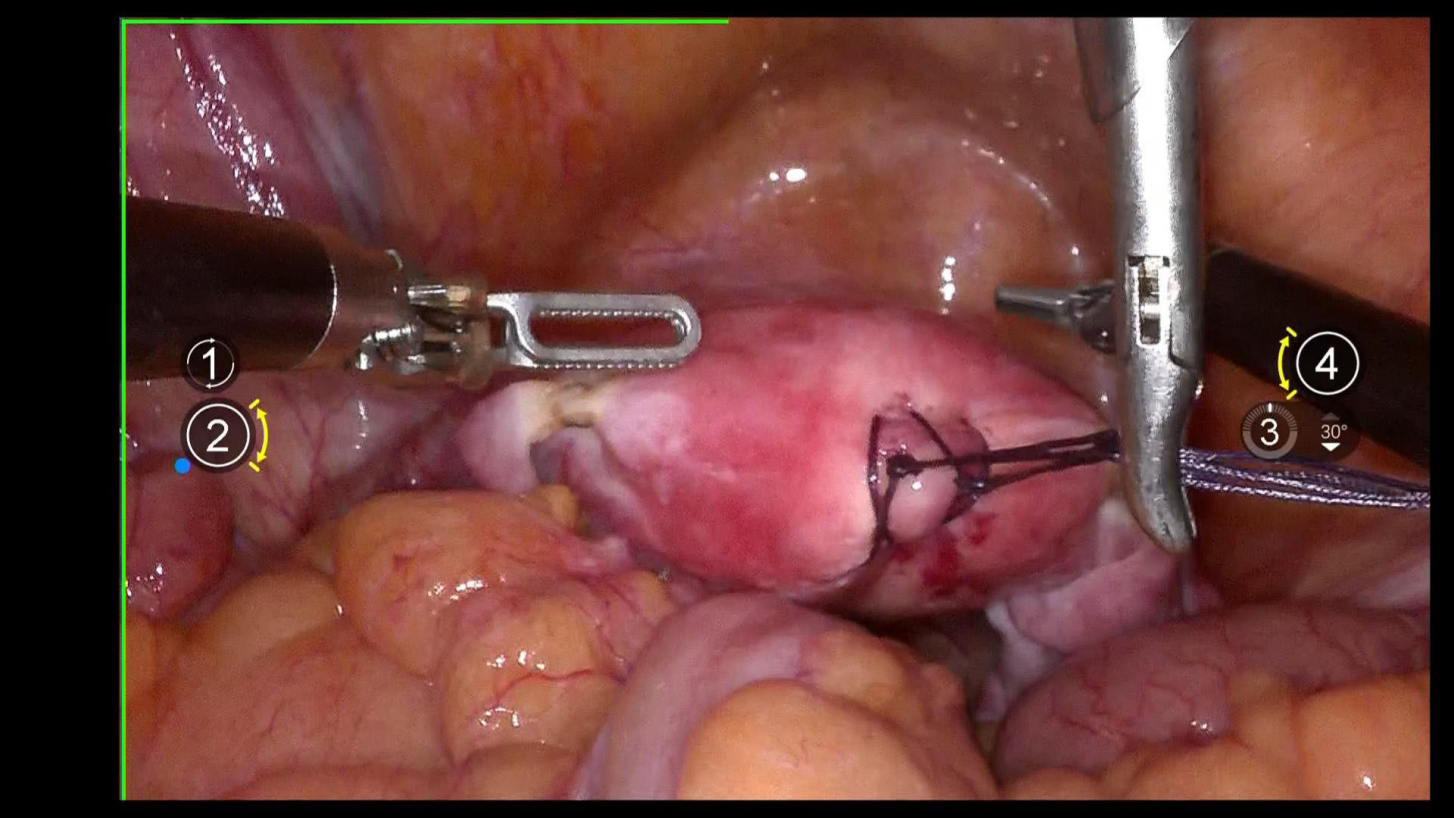
Shows uterine suspension.

Using 1–0 absorbable suture, two “8” stitches are placed on the posterior wall near the uterine fundus. The uterus is manipulated using a laparoscopic needle holder, which grasps the uterine sutures, replacing uterine manipulation by a uterine manipulator to avoid compression of the tumor and tumor spillage. (**Figure 2**).

### 4.3 Lymphrectomy

**Figure 3:**
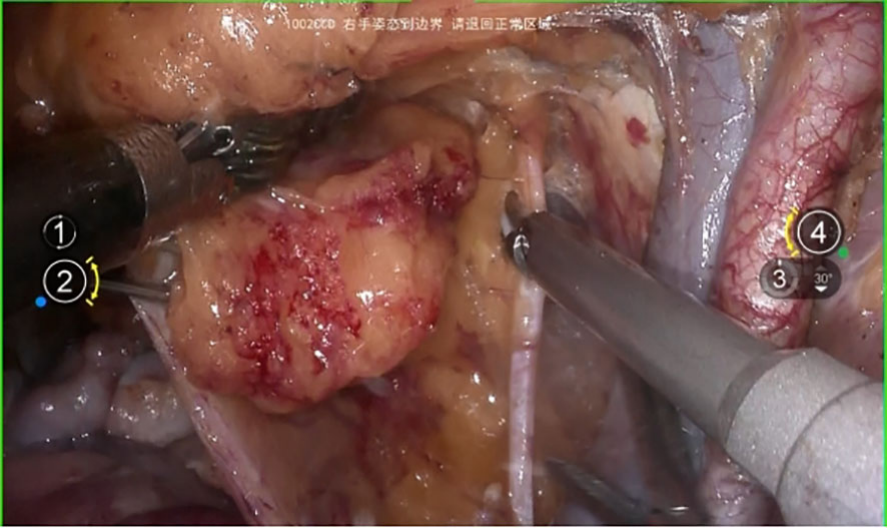
Systematic pelvic lymphadenectomy.

The surgical procedure began with an incision of the peritoneum covering the psoas major muscle to expose the iliac vessels. Next, the peritoneum was carefully opened along the abdominal aorta in a cephalad direction, allowing full exposure of the aortic bifurcation, the iliac vascular region, and the ureter. Following this, complete resection of the presacral and pelvic lymph nodes was performed from the caudal aspect of the aortic bifurcation. During this lymph node dissection, the pelvic floor fascia beneath the obturator nerve was clearly exposed dorsally, the lumbosacral trunk and iliac vein were identified laterally, and the medial boundary was defined external to the internal iliac artery and vein. (**Figure 3**).

### 4.4 Lateral parametrium

**Figure 4:**
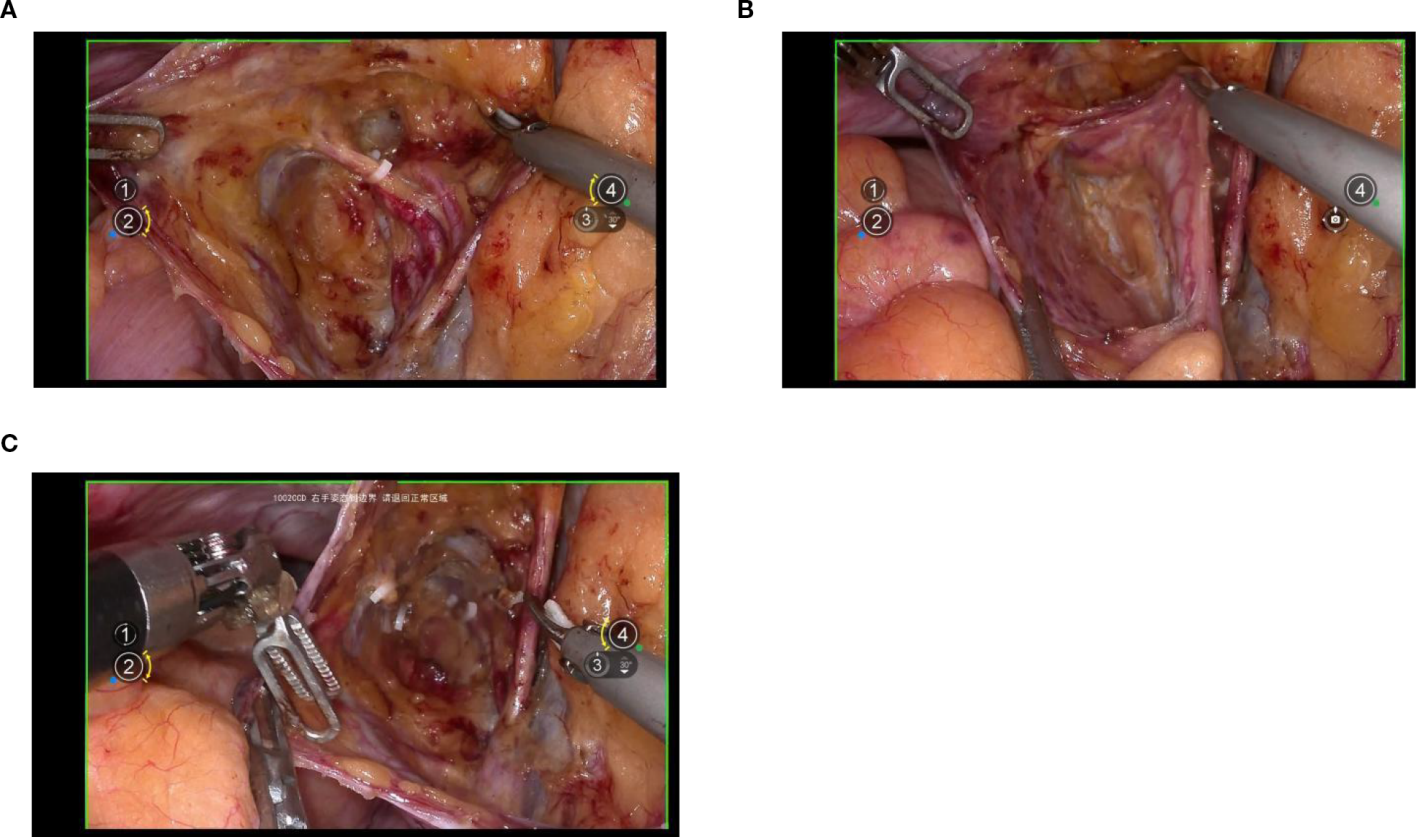
**(A, B)** shows the separation of the ureteric embryonic compartment, hindgut embryonic compartment, urogenital embryonic compartment, and Müllerian embryonic compartment by opening the paravesical space, Latzko’s pararectal space, and Okabayashi’s pararectal space; **(C)** shows the cut ends of the uterine artery and the deep uterine vein.

Exposure of the lateral parametrium is primarily achieved by separating the paravesical space, Latzko’s pararectal space, and Okabayashi’s pararectal space. This allows for the dissection of the ureteric embryonic compartment, the mesorectum of the hindgut embryonic compartment (lateral rectal wall), the posterior lateral bladder wall of the urogenital embryonic compartment, and the mesometrial outlet of the Müllerian embryonic compartment. Following vascular clamping between the Latzko’s pararectal and paravesical spaces, the uterine artery, superficial uterine vein, and deep uterine vein are sharply dissected. (**Figures 4A-C**).

### 4.5 Dorsal parametrium

**Figure 5:**
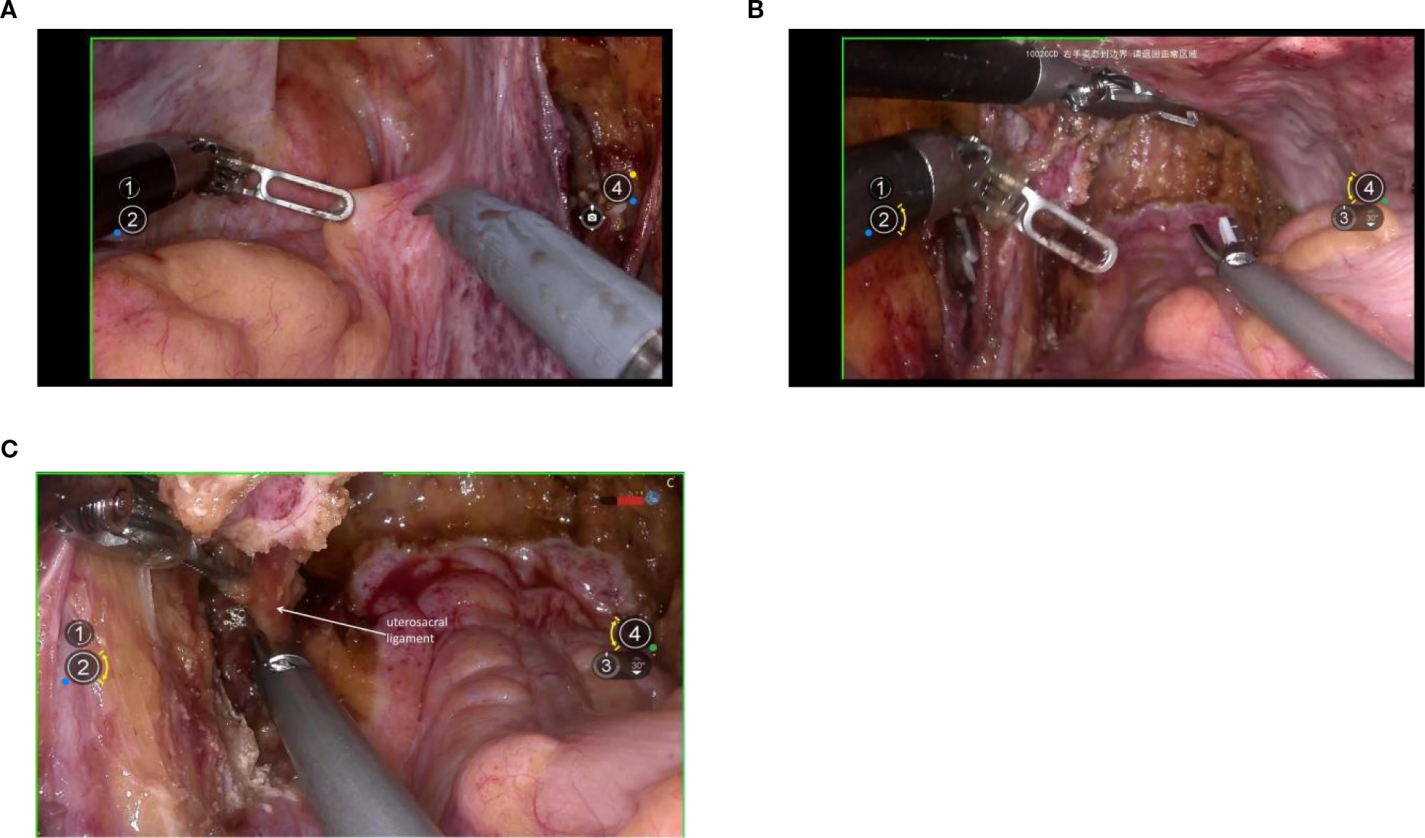
**(A)** shows the membranous bridge between the hindgut embryonic compartment and the Müllerian embryonic compartment; **(B)** shows the separation of the hindgut embryonic compartment and the Müllerian embryonic compartment; **(C)** shows the transection of right uterosacral ligament.

Exposure of the dorsal parametrium is achieved by opening the membrane bridge (15–17) between the rectum of the hindgut embryonic compartment, the posterior vaginal wall of the Müllerian embryonic compartment, and the sacral ligaments. This allows access to the rectovaginal space, facilitating the separation of the hindgut embryonic compartment from the Müllerian embryonic compartment. The sacral ligaments should be sharply dissected at the level of the sacral fascia. **Figures 5A-C**.

### 4.6 Ventral parametium

The management of the ventral parametrium presents considerable complexity due to the involvement of various embryonic compartments. Specifically, it encompasses structures from the urogenital embryonic compartment, including the bladder, and from the ureteric embryonic compartment, the ureters. Additionally, it involves multiple structures from the Müllerian embryonic compartment, such as the cervix, vagina, parametrium, and paravaginal connective tissue.

**Figure 6:**
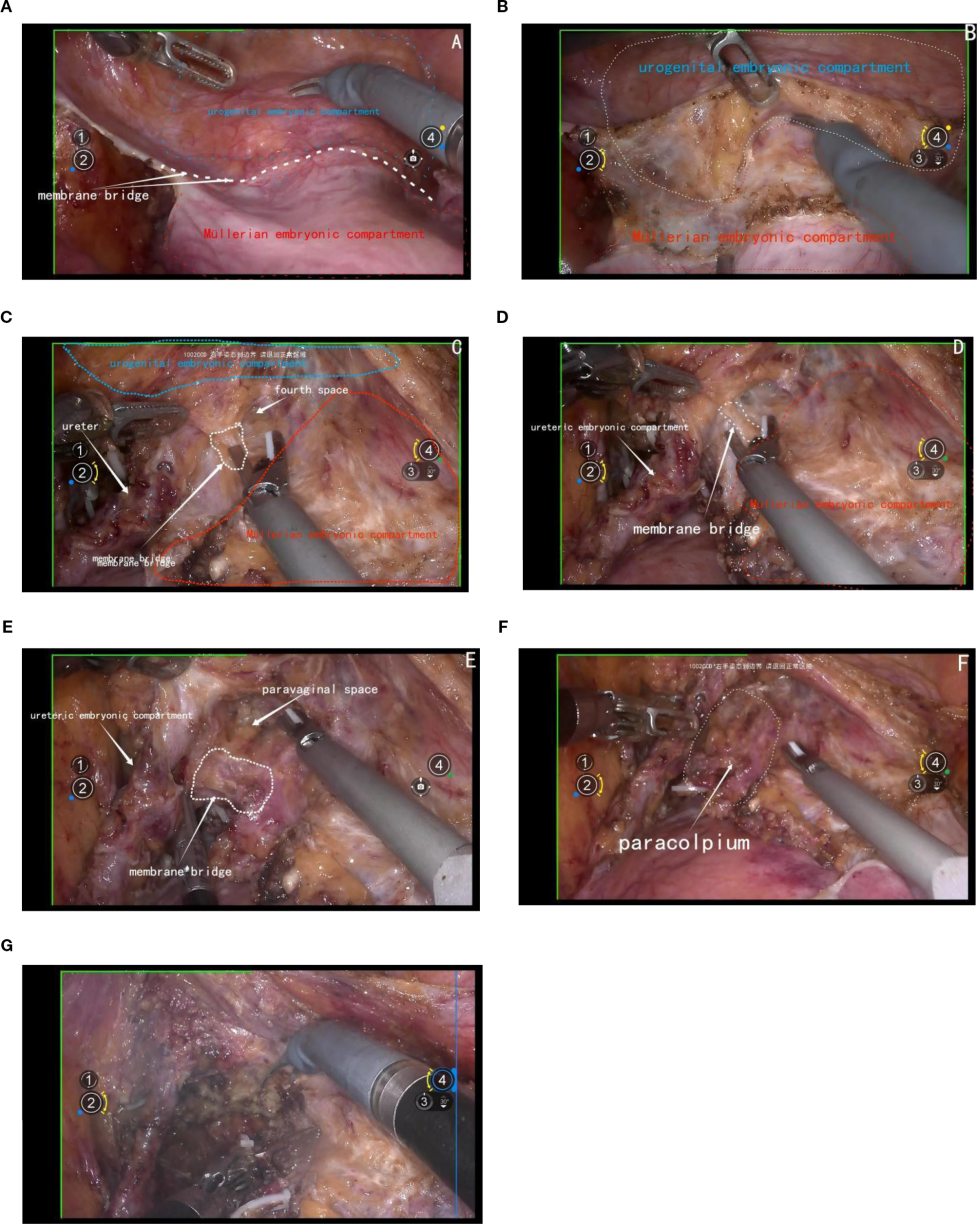
**(A)** shows the membrane bridge between the urogenital embryonic compartment and the Müllerian embryonic compartment; **(B)** shows the separation of the urogenital embryonic compartment and the Müllerian embryonic compartment; **(C)** shows the vesicocervical ligament, which serves as the membrane bridge connecting the bladder of the urogenital embryonic compartment and the parametrium of the Müllerian embryonic compartment; **(D)** shows the membrane bridge connecting the ureteric embryonic compartment and the Müllerian embryonic compartment; **(E)** shows the membrane bridge, known as the vesicovaginal ligament between the posterior bladder wall of the urogenital embryonic compartment and the paracolpium of the Müllerian embryonic compartment; **(F)** shows the exposure of the paracolpiium after the transection of the vesicovaginal ligament; **(G)** shows the transection of the paracolpium.

The initial step involves opening the membrane bridge between the urogenital and Müllerian embryonic compartments, known as the bladder peritoneum reflection. This maneuver exposes the vesicocervical space and vesicovaginal space. Subsequent lateral widening of the vesicovaginal space reveals the fourth space. At this point, the vesicocervical ligament, which serves as the membrane bridge connecting the bladder of the urogenital embryonic compartment and the parametrium of the Müllerian embryonic compartment, becomes evident. Following transection of this ligament, the membrane bridge connecting the ureteric embryonic compartment and the Müllerian embryonic compartment can be identified. Resection of this membrane bridge exposes the paravaginal space, and subsequently, complete exposure of the membrane bridge, known as the vesicovaginal ligament between the posterior bladder wall of the urogenital embryonic compartment and the paracolpium of the Müllerian embryonic compartment is achieved. Subsequent dissection of the paravaginal connective tissue parallel to the fascia of the levator ani muscle ensures thorough removal of the abdominal parametrium of the Müllerian embryonic compartment. **Figures 6A-G**.

### 4.7 Closure of the vagina

**Figure 7:**
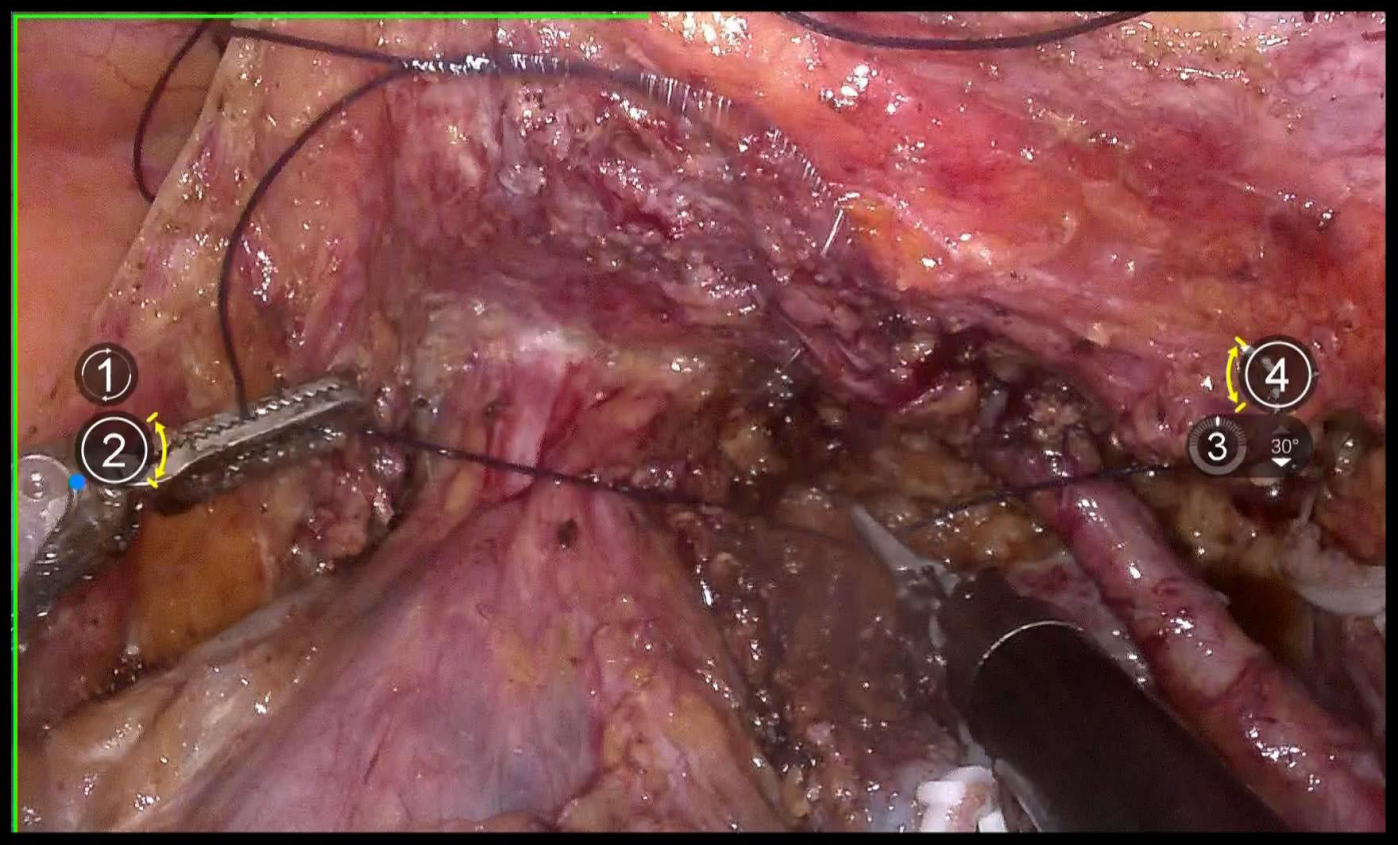
shows the closure of vagina with absorbable suture.

Use 1–0 barbed suture for continuous circular suturing of the vaginal wall to close the vagina. Ensure that during circular incision of the vagina, the cervical tumor does not spill out(**Figure 7**). It should be noted that the suture should be placed just caudal to the level of paracolpium transection. During suturing, the assistant must provide direct visual confirmation via the vaginal route, with particular attention to ensuring complete vaginal closure.

### 4.8 Flush and transect the vagina

**Figure 8:**
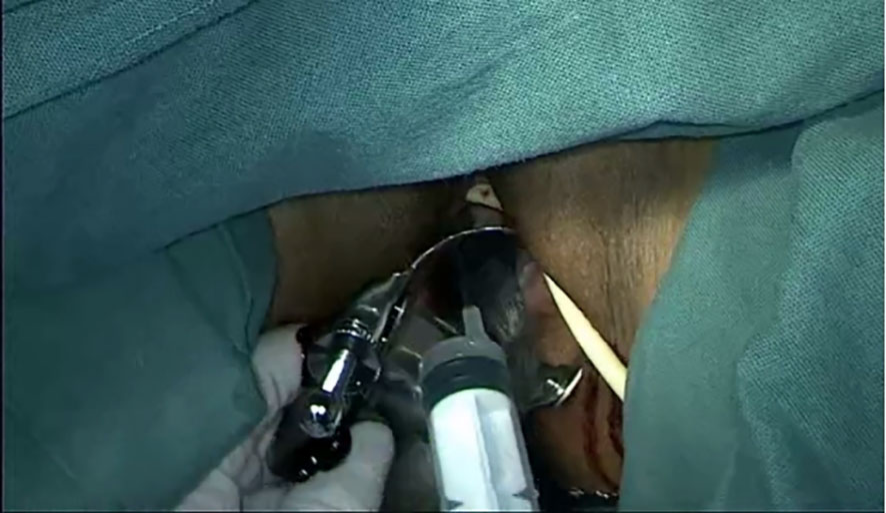
Shows the flush of vagina.

**Figure 9:**
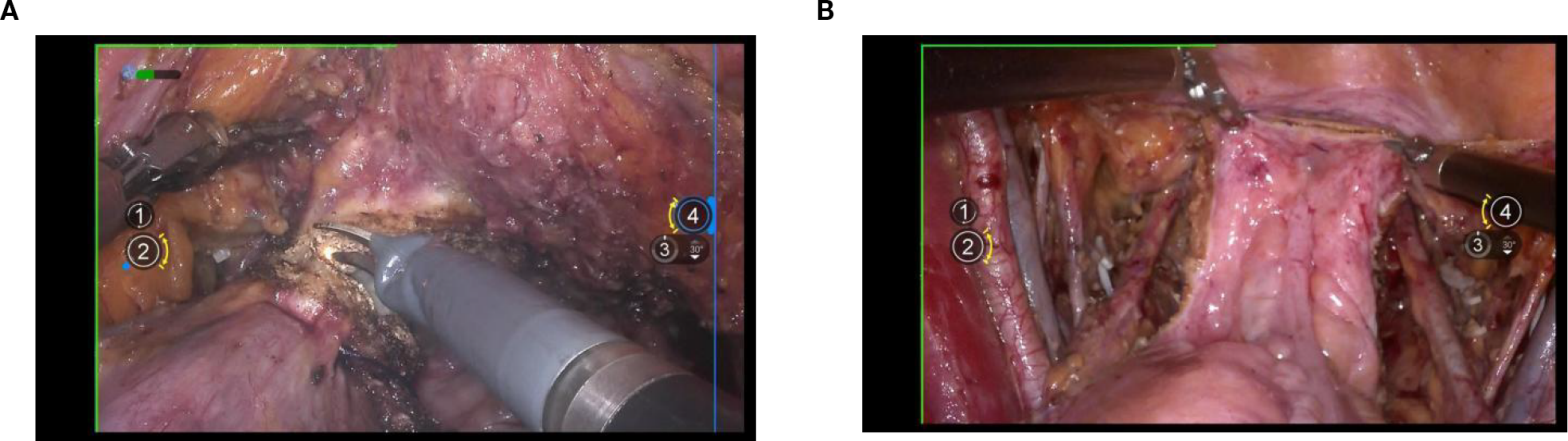
**(A)** shows the incision of vagina; **(B)** shows the closure of vagina.

Repeatedly irrigate the vagina with sterile distilled water at 42°C to dislodge any tumor cells adhering to the vaginal wall, thereby preventing the entry of tumor cells into the wound during circular incision of the vaginal wall(**Figure 8**). Then, transect the vagina with a monopolar and stump the vaginal with absorbable suturesl(**Figures 9A, B**).

## 5 Case presentation

The first patient managed by our team undergoing robot-assisted total Müllerian compartment resection (TMCR) was a 63-year-old postmenopausal woman who presented to the gynecology outpatient clinic with a chief complaint of vaginal bleeding following sexual intercourse. Gynecological examination identified a cauliflower-like cervical mass measuring 1.5 cm × 1.0 cm × 1.0 cm. Doppler ultrasound demonstrated abundant blood flow signals within the lesion, raising a high suspicion of cervical malignancy. To confirm the diagnosis, human papillomavirus (HPV) testing was performed, which revealed HPV type 16 positivity. Histopathological analysis of the cervical biopsy specimen confirmed the diagnosis of cervical squamous cell carcinoma. Computed tomography (CT) and magnetic resonance imaging (MRI) both demonstrated a cervical mass approximately 1.5 cm in diameter, with no evidence of parametrial involvement or lymph node metastasis. Based on the patient’s clinical history, physical examination, and auxiliary diagnostic findings, a preoperative diagnosis of stage IB1 cervical squamous cell carcinoma was established. Prior to surgery, the patient and her family were thoroughly informed about the available treatment options and the implications of the LACC trial. Considering the advantages of minimally invasive surgery, the patient elected to undergo robot-assisted TMCR. The procedure lasted 180 minutes, with an estimated intraoperative blood loss of 100 ml. Bowel function resumed within 48 hours postoperatively, and the pelvic drainage tube was removed on postoperative day 4. The urinary catheter was removed three weeks after surgery, and no postoperative complications or urinary dysfunction were observed during the follow-up period. Final histopathological evaluation revealed moderately differentiated squamous cell carcinoma with no lymphovascular space invasion (LVSI) and a stromal invasion depth of less than one-third of the cervical wall thickness. No adjuvant radiotherapy or chemotherapy was administered postoperatively. The patient has been followed up for 46 months and remains in good general health with no evidence of disease recurrence.

## 6 Discussion

Membrane anatomy is a concept that integrates the understanding of tissue planes and embryologic compartments. The female reproductive tract, including the uterus, cervix, and vagina, their neurovascular supply structures, and a coat of condensed connective tissue (18), develops from distinct embryonic compartments. These compartments include the Müllerian embryonic compartment, the ureteric embryonic compartment, the urogenital embryonic compartment, and the hindgut embryonic compartment. Each compartment is enveloped by a specific membrane, facilitating the dissection and isolation of these structures during surgery.

The local spread of a malignant solid tumor is generally considered a random process that follows paths of least mechanical resistance. However, we have proposed that, until the later stages of disease progression, local tumor spread is directed by positional cues provided by the mesenchyme within the anatomical morphogenetic unit of the originating organ or tissue (19). Patients with a high risk of local recurrence are given adjuvant radiotherapy following an R0 resection, based on the tumor’s histopathological characteristics, which is the standard treatment protocol for carcinoma of the uterine cervix in FIGO stages IB–IIA. However the incidence of treatment-related morbidity is elevated when surgery is combined with adjuvant radiotherapy compared to radiotherapy alone, yet both approaches achieve at least comparable locoregional tumor control (20), so the radical hyesterctomy is not real radical. Professor Höckel significantly improved the oncological outcomes of cervical cancer patients by performing embryonic compartment-based radical hysterectomy guided by membrane anatomy (3).

Alongside traditional laparoscopy, the use of robot-assisted laparoscopic surgery in gynecology has grown substantially. Some studies have highlighted the safety and efficacy of robotic surgery in the treatment of cervical cancer (12). The findings of the LACC trial indicated that minimally invasive surgery for cervical cancer is associated with poorer oncological outcomes and a higher incidence of complications. However, due to factors related to surgical trauma and other clinical considerations, minimally invasive procedures may still be performed when patients make informed decisions in favor of such approaches. In recent years, the techniques used in minimally invasive surgery have evolved beyond those included in the LACC trial criteria. These advancements include a greater emphasis on avoiding uterine manipulation and on performing vaginal cuff closure prior to vaginal transection. Such refinements in surgical technique have shown a positive impact on oncological outcomes (21).

The approach for performing a radical hysterectomy is ultimately determined by the surgeon’s experience. Following the LACC trial findings, abdominal surgery is currently the preferred method for treating early-stage cervical cancer. Retrospective studies consistently indicate lower pregnancy rates following abdominal radical trachelectomy compared to vaginal radical trachelectomy or minimally invasive techniques. Interestingly, robot-assisted radical trachelectomy has shown the highest pregnancy rates, though larger studies are needed to confirm these findings.

A meta-analysis by Marchand G et al. (22)compared laparoscopic radical hysterectomy (LRH) and open radical hysterectomy (ORH) in early-stage cervical cancer, excluding robotic-assisted cases (RRH). Across 60 studies and 42,994 patients, no significant differences were found in 5-year overall survival (OR = 1.24, P = 0.12), disease-free survival (OR = 1.00, P = 0.98), recurrence (OR = 1.01, P = 0.95), or intraoperative complications (OR = 1.38, P = 0.10). LRH reduced blood loss (MD=-325.55, P<0.001), transfusions (OR = 0.28, P = 0.002), postoperative complications (OR = 0.70, P = 0.005), and hospital stay (MD=-3.64, P<0.001). ORH was faster (MD = 20.48, P = 0.007) and removed more lymph nodes (MD=-2.80, P = 0.004). The study concluded that LRH is safe and effective for early-stage cervical cancer with better short-term outcomes. Song et al. (23) compared minimally invasive radical hysterectomy (MIRH) with and without protective vaginal ring resection. Findings showed that adding the ring resection improved MIRH outcomes, making them comparable to open surgery for early cervical cancer. Wenzel et al. (24) compared LRH and ARH. Adjusted analyses found no significant differences in 5-year DFS (89.4% vs 90.2%, HR = 0.92) or OS (95.2% vs 95.5%, HR = 0.94), with consistent results across tumor sizes. An Indian retrospective study (25) compared RRH and ORH. No significant differences were found in 3- or 5-year DFS or OS. RRH, however, had less blood loss (100 ml vs 300 ml, P<0.001), shorter surgery (162.5 vs 180 min, P = 0.005), and shorter hospital stay (3.9 vs 6.3 days, P<0.001). Tanitra Tantitamit et al. (26) found no significant differences in 5-year OS (RR = 1.0, 95% CI 0.98–1.03, p=0.33) or DFS (RR = 1.02, 95% CI 0.97–1.06, p=0.98) between LRH and ARH. Pathological risk factors were similar. LRH reduced blood loss, transfusions, complications, and hospital stay. Our study followed 20 robotic TMCR patients for 18 months (15), no recurrence or cervical cancer deaths were observed. Another study on 55 cases of TMCR surgery (27) showed that all patients achieved R0 resection, with no intraoperative or severe postoperative complications. The median blood loss was 72 mL, the median operation time was 282 minutes, and the median follow-up was 15 months without recurrence. Moreover, the bladder and intestinal functions recovered well after the operation.Although these studies were conducted relatively late, these results support that minimally invasive surgery, compared with open surgery, can provide non-inferior short-term oncological outcomes and better perioperative recovery.

Robotic radical hysterectomy offers several advantages over laparotomy for early-stage cervical cancer patients. These benefits include reduced blood loss, a lower need for blood transfusions, fewer complications, and shorter hospital stays, despite the longer operative times.

## 7 Conclusion

The application of this technique requires a high level of expertise and familiarity with embryologic compartments and membrane anatomy. It also demands the use of advanced surgical tools and technologies, such as robotic assistance, to achieve the necessary precision and control, The adoption of precise surgical techniques helps standardize and optimize procedures, thereby facilitating the learning process.

Membrane anatomy and embryonic compartment-based radical hysterectomy represent a paradigm shift in the surgical treatment of cervical cancer. This approach not only enhances the precision and safety of the procedure but also contributes to improved oncologic outcomes by preventing tumor spillage and reducing postoperative complications. As surgical techniques continue to evolve, the integration of embryologic principles will play a pivotal role in advancing gynecologic oncology.

## Data Availability

The original contributions presented in the study are included in the article/supplementary material. Further inquiries can be directed to the corresponding author.
